# Stem Cell Therapy for Diabetic Foot Ulcers: Theory and Practice

**DOI:** 10.1155/2022/6028743

**Published:** 2022-12-06

**Authors:** David Lubasi Nalisa, Md. Moneruzzaman, Geoffrey J. Changwe, Youchaou Mobet, Li Ping Li, Yu Jin Ma, Hong Wei Jiang

**Affiliations:** ^1^Department of Metabolism and Endocrinology, Endocrine and Metabolic Disease Center, The First Affiliated Hospital and College of Clinical Medicine of Henan University of Science and Technology, Luoyang, China 471003; ^2^Medical Key Laboratory of Hereditary Rare Diseases of Henan, Luoyang, China 471003; ^3^Luoyang Sub-Center of National Clinical Research Center for Metabolic Diseases, Luoyang, China 471003; ^4^Department of Rehabilitation Medicine and Physiotherapy, Qilu Hospital of Shandong University, Shandong University, Jinan, Shandong 250012, China; ^5^Department of Cardiovascular and Thoracic Surgery, National Heart Hospital, Off-Airport Road, 10101 Lusaka, Zambia; ^6^Department of Obstetrics and Gynecology, The Third Affiliated Hospital of Chongqing Medical University, Chongqing 401120, China

## Abstract

Diabetic foot ulcers are associated with increases in limb amputation, morbidity, and mortality. Recently, a stem cell application is emerging as promising adjuvant therapy. We presented available remedies by conducting a literature review on the application, safety, and efficacy of stem cell therapy. Relevant literature, including randomized control trials and article journals, was obtained from reputable search engines (PubMed, Scopus, and Web of Science). We analyzed five credible cohorts, with variable sources of stem cells, in a total of 216 participants, 151 males and 65 females, age (mean ± SD) of 64.5 ± 9.6 years. With an average success of 86.41% in all Wagner-II lesions, mesenchymal SCA (stem cell application) is safe and effective, hence can significantly prevent limb amputation.

## 1. Introduction

An open sore or wound on the foot of a subject with established diabetes mellitus (DM) is commonly referred to as a diabetic foot ulcer (DFU). The development of DFU is attributed to hyperglycemia, which leads to poor circulation (peripheral artery disease) and loss of feeling (neuropathy), therefore, promoting wound development and impairing the healing process [1]. It is reported that about 15% of patients with DM develop DFUs, and of those, 14 to 24% undergo limb amputation. Besides the burden of medical expenditure and chronic morbidity, DFU significantly contributes to early mortality, as 68% of amputees succumb within five years due to sepsis and mental stress [2].

Because DM affects several interconnected physiological systems equally, the management of its complications, thus, DFU calls for a multidisciplinary approach using several conventional therapies, which are well discussed in appropriate sections and emerging treatment options to reduce both disease burden and cost [3]. In the past two decades, the medical world has experienced tremendous development in various fields, including imaging to determine microcirculation and stem cells to facilitate regeneration [4]. Those as mentioned above and the poor outcomes of DFU management have compelled clinicians, physicians, and diabetologists to seek the option of utilizing stem cells as an adjuvant therapy to either rekindle the loss or amplify the diminishing healing process.

It is from the background above that this project seeks to elucidate the available DFU treatment options and further enlighten on the potency, efficacy, drawbacks, and perspectives of stem cell therapy in the management of DFU.

## 2. Literature Selection and Screening

**Figure 1:**
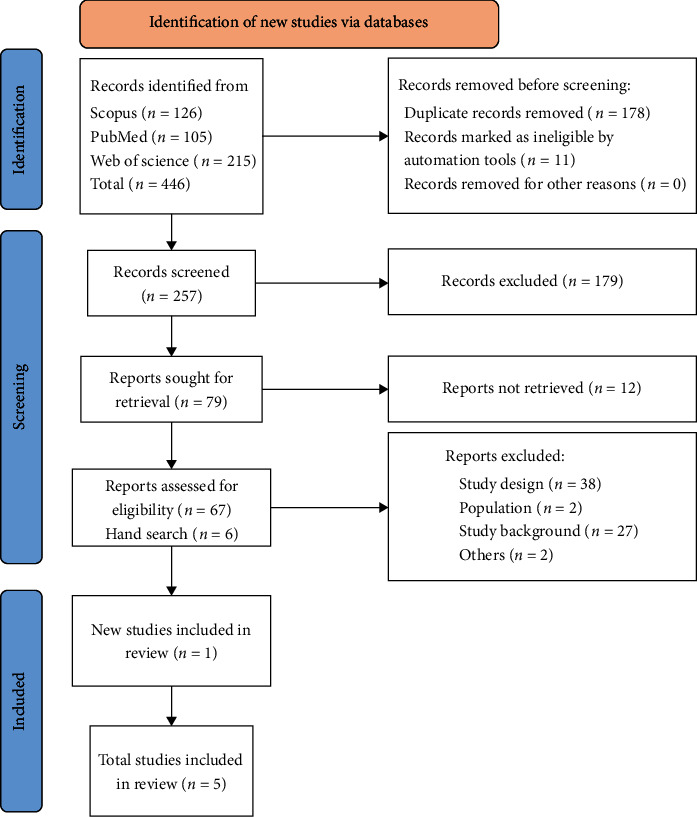
Selection process of studies regarding diabetic foot lesions and stem cell therapy. Shows the process of study selection that includes identification and screening until the final inclusion of data for review.

To identify relevant literature, we developed a search strategy to obtain data from PubMed, Web of Science, and Scopus for this review project. The search strategy included original research journal articles and randomized control trials (RCTs), all published in English. Based on PRISMA principles [5] (Table 1), we obtained all literature mainly focusing on the management of DFU published in the fields of endocrinology and internal medicine. An independent author performed selection, confirmation, curation, and initial analysis of the literature for consideration. (Figure 1) Only literature published from 2003 to May 2021 was considered for review.

## 3. Role of Stem Cells and Other Treatments for Diabetic Foot Ulcers

Despite the realization and formation of multidisciplinary units to manage, the burden due to foot ulcers in patients with established DM remains higher than the desired level [1, 2]. DFU as a complication of DM has been managed using a variety of conventional approaches with variable outcomes. Furthermore, available therapies are not readily accessible in many resource-poor nations due to a lack of consumables and specialized personnel. Although the available treatment options have contributed immensely, the higher morbidity and mortality rate has compelled the medical world to seek stem cells as an adjuvant therapy to conventional approaches [4]. It is worth pointing out that stem cell therapy emanates from various fields, such as neurology and hepatology. Reports of successful regeneration of the tissues in question had been achieved [6]. The success of stem cell therapy in the management of DFU has a rather foggy history, consensus, efficacy, and prospects. Having analyzed adequate literature regarding this topic, it is worthy of highlighting the available treatment options once again as follows:

### 3.1. Health Education

Information, education, and communication (IEC) may be viewed as a silent therapy that is not only applicable to subjects with DM and potentially DFU but cuts across a variety of noncommunicable ailments. Prevention of trauma and subsequent development of DFU can be achieved by educating the subject and caregivers on the importance of foot care, nail care, and the use of right-sized footwear. Although IEC is mainly conducted by junior health personnel, occasionally, a senior health practitioner should be able to conduct a session to appreciate the concerns. Furthermore, patients tend not only to value and appreciate but also to improve compliance when a senior physician delivers IEC [7]. A well-packaged IEC-delivered periodically in a systematic manner would help subjects understand and appreciate the role of etiology in developing DFU with potential limb amputation [1, 2].

### 3.2. Multidisciplinary Approach

Having realized that DM affects various physiological systems, it has become a standard to form specialized units to prevent and manage DFU in subjects with DM [3]. A specialized unit, in most cases, is composed of a diabetologist, endocrinologist, orthopaedist, vascular surgeon, physiotherapist, nutritionist, psychologist, and adequate nurses specialized in DM [6, 8]. A multidisciplinary approach is rendered incomplete without the involvement of the immediate caregiver or family members through periodical IEC.

### 3.3. Comorbidity Management

Control of metabolic status has been cited by most literature as a cardinal step in the control and management of DM and associated comorbidities [9]. To achieve timely wound healing, it is vital to optimize blood sugar control to prevent its negative effect on cellular immunity and subsequent infection. Evidence shows that optimal glycemic control does not only impede the onset but hinders the development of retinopathy, nephropathy, and above all, neuropathy, the condition responsible for the development of DFU [10]. Recent guidelines (IWGDF 2019) recommend the application of insulin, if necessary, to optimize glycemic control and further treat edema or malnutrition if present [11]. In one of the studies, intensive glycemic control was associated with a statistically significant reduction in the risk of amputation in DFU (RR, 0.56; 95% CI 0.45-0.94) compared to less intensive control [12].

### 3.4. Foot Infection Management

Prevention of any infection in a DM subject is cardinal in maintaining optimal metabolic status and glycemic control. In the absence of neuropathy, signs and symptoms of established infection, such as tenderness, warmth, induration, and erythema, sound a warning and compel the subject to seek medical attention [2]. However, the opposite is true among DM subjects with DFU with already established neuropathy. In this era of progressive antimicrobial resistance, antimicrobials are applied to eliminate infections and not heal the lesions. The International Working Group on Diabetic Foot advocates for limited usage of antibiotics by establishing guidelines and severity classifications. Despite the decades of studying DFU, the lesion remains a medical challenge that demands more exploration for timely and definitive treatment options [10, 11, 13].

## 4. Established Therapies for Diabetic Foot Ulcers

Established DFU, as defined above and in literature [11], calls for standard care to alleviate morbidity and avoid possible limb amputation. In an ideal medical setup, care involves the following stages:

### 4.1. Wound Dressing

Good wound dressing must protect the ulcer from the external environment, thus preventing possible infection, relieving symptoms, and facilitating healing. In this regard, wound dressing constitutes a part of the treatment in DFUs. Depending on the physiology of the lesion, numerous types of wound dressing are currently in existence [14]. Nevertheless, wound dressing must be taken as an adjuvant therapy as it cannot substitute medically proven curative approaches. Moreover, currently, no single wound-dressing product achieves the physiological needs for proper healing of DFU [15]. The gold standard for the treatment of diabetic foot ulcers comprises wound debridement, control of infection, revascularization where indicated, and ulcer off-loading. The “sharp method” of debridement is distinctly one of the gold standards in aiding wound healing, notably favoring the healing process of wounds, including diabetic foot ulcers. Regular food examination, patient education, simple hygiene practices, the provision of appropriate footwear, and the prompt treatment of minor injuries can reduce the occurrence of ulcers by 50% and eliminate the need for major limb amputation in nonischemic limbs. Without concurrent management of ischemia, infection, and adequate off-loading, no known therapies would be effective [16, 17].

### 4.2. Negative Pressure Wound Therapy

Negative pressure wound therapy is a consequence of recent medical technological advancements in wound dressing and care. It involves the use of a wound dressing fitted with a vacuum machine, which is adjusted accordingly to automatically suction tissue fluids into a canister. The tissue fluids contain bioactive substances such as inflammatory cytokines and proteases, both of which contribute to wound healing negatively. In addition, if left untouched, suctioned tissue fluids or exudates provide a fertile ground for the growth of infectious pathogens, thus posing a danger for the development of sepsis. The use of negative pressure wound therapy is believed to reduce the frequency of wound dressing and, consequently, prevent unnecessary exposure to the environment [18, 19]. Nevertheless, Mohseni et al. did not agree that this gadget was as effective as has been reported [20].

### 4.3. Hyperbaric Oxygen Therapy

In a lesion of DFU, infection promotes local hypoxia, which promotes the growth of anaerobic pathogens, impairs local tissue perfusion, and consequently triggers cell death and necrosis. The use of hyperbaric oxygen therapy (HBOT) is believed to improve local tissue perfusion, and the improved perfusion stimulates the production of growth factors, collagen synthesis, and neovascularization, therefore, accelerating wound healing [21–24]. In addition, HBOT retains a bactericidal effect against anaerobic pathogens, therefore, reducing the indiscriminate use of antibacterial drugs [22, 23]. Despite the reported benefits, HBOT has few adverse effects, including toxicity and noncompliance [24].

### 4.4. Off-Loading

The basic principle of off-loading depends on reducing excessive pressure that is prone to compromise the regenerating tissue [15, 25]. Increased plantar foot pressure is one of several key factors that lead to diabetic foot ulcers. Off-loading has an important role in promoting wound healing and preventing foot ulceration in diabetes [25]. Based on research evidence, Total Contact Casting bracing (TCC) has been considered the gold standard interventional approach for DFU off-loading. Other devices include bracing, walking aids, and therapeutic footwear [26].

### 4.5. Wound Debridement

Wound debridement is a procedure that involves the removal of nonviable or infected wound tissues to provide a conducive environment for the regeneration of healthy tissue. Depending on the tissue type, debridement can be accomplished using surgical, autolytic, or biological techniques [27]. However, as mentioned earlier, these three techniques lack quality and comparative effectiveness due to imprecision and methodologic limitations, as observed in one meta-analysis study [26]. Consequently, available expertise, clinical context, procedural cost, and patient choice dictate the choice of the debridement method [28].

#### 4.5.1. Challenges on Diabetic Foot Ulcer and Healing Process

**Figure 2:**
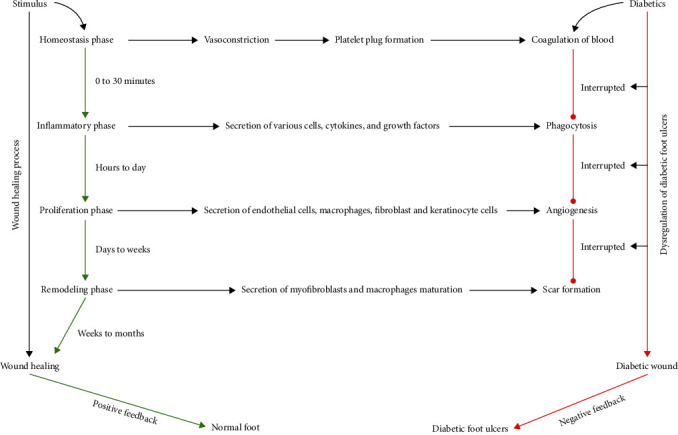
Mechanism of wound healing and dysregulation of diabetic foot ulcers. The stages of wound healing and dysregulation of diabetic foot ulcers: Wound healing begins with hemostasis phase, where a platelet plug prevents blood loss and a preliminary fibrin matrix is formed. Inflammation phase ensues to avoid infection (phagocytosis), with the secretion of neutrophils, macrophages, cytokines, and growths factors to remove cell debris. During the proliferative phase, endothelial cells, macrophages, fibroblast, and keratinocytes cells accelerate angiogenesis by closing the wound gap and replacing the initial fibrin clot with granulation tissue. Finally, the remodeling phase causes overall wound contraction by secretion of myofibroblasts and macrophages maturation. In diabetic wounds, active diabetes interrupts routine wound-healing phases, leading to poor outcomes.

Wound healing is an aggressive-convoluted process that gets established in a quadriphasic whenever tissue integrity is compromised [8]. Ideally, normal stages of healing materialize in acute wound settings where pronounced indicators of healing are observed within 30 days. In DM subjects, metabolic disturbances, infections, and occlusive vascular lesions derail the timely transition of wound healing phases by prolonging or locking in a particular phase, thus leading to chronicity or lesion enlargement [29] (Figure 2).

## 5. Theory of Stem Cell Therapy (SCT)

To understand the current dynamics, limitations, and successes of SCT during the stem cell therapy era, from 1930 to 1998, a plethora of developments in medicine unfolded. Of note, concurrent with progressive developments in the field of stem cell therapy (SCT) is the monumental developments in the field of human medical imaging. The inception of echocardiography-computed tomography and magnet resonance imaging has revolutionized the diagnosis and follow-up strategies of a myriad of somatic and systemic lesions [30]. Towards the end of the previous century, the isolation of embryonic pluripotent stem cells brought much anticipation and excitement to the SCT field. Unfortunately, this breakthrough has not translated into meaningful application due to backlash from religious and ethical groupings. Consequently, at present, adult stem cells remain a cornerstone of SCT (Table 2).

### 5.1. Effects of Stem Cells in Preventing Major Limb Amputations

Aiming to prevent major limb amputations in diabetic patients suffering from critical limb ischemia and foot ulcer using local application of autologous bone marrow concentrates, Procházka et al. randomized subjects into AB-MSC concentrates prepared as BMC treatment group I and (standard treatment) control group II. The primary endpoints were assessments based on clinical evaluation. These included major limb amputation, degree of pain, and function were evaluated before treatment at day 0, and after that, at day 90 and day 120 of clinical follow-up intervals. As expected, the results demonstrated improved clinical outcomes. The frequency of significant limb amputations was significantly lower in the treatment group compared with the control group. In the treatment group, major limb amputation was only noticeable in those subjects suffering from severe lymphopenia and thrombocytopenia in BMC at treatment time. In relation to the control group, the treatment group reported all AB-MSC subjects, completed clinical follow-up, and showed positive outcomes by day 90. Simultaneously, the control group had recorded none until day 120. Using the EQ-50 quality of life questionnaire, the treatment group results revealed subjective improvement in ischemic pain within three days of a stem cell application. On the contrary, patients with healing limbs encountered long-standing pain of up to half a year. As a result, the authors pointed out a possible mechanism where multicellular treatment, precisely bone marrow concentration, promotes wound healing [31].

### 5.2. Impact of Bone Marrow Mesenchymal Stem Cells and Bone Marrow-Derived Mononuclear Cells

In an effort to determine preferable stem cells for the treatment of critical limb ischemia and foot ulcer in diabetic patients, Lu et al. [32] randomized two groups of autologous Bone Marrow Mesenchymal Stem Cells (BM-MSCs) and Bone Marrow Mononuclear Cells (BM-MNCs) and administered intramuscular injections. Ex vivo expanded human BM-MSCs were distinguished by their ability to proliferate in culture, their fusiform shape, the presence of mesenchymal antigens CD29, CD71, CD90, and CD105, and the absence of myeloid surface antigens CD45 and CD34. The target number of ex vivo expanded BM-MSCs was achieved in all patients after three passages or three days after the primary bone marrow aspiration. In comparison, BM-MNCs came in a variety of shapes, mostly circular or semicircular, with a wide range of sizes. The desired number of BM-MNCs was obtained immediately after density gradient centrifugation of the primary bone marrow. To prepare enough BM-MNCs for transplant, at least a 3-hour systemic/epidural anesthesia was required, as well as aspiration of large amounts of marrow (300-500 ml). Nevertheless, many patients were unable to tolerate the BM-MNC cytotherapy. During this trial, only 30 ml of bone marrow under local anesthesia was required to achieve the desired number of BM-MSCs, and 300 ml of bone marrow under epidural anesthesia was required to achieve the desired number of BM-MNCs. BM-MSCs and BM-MNCs are known to secrete angiogenic factors, though, under various experimental conditions, protein levels of angiogenic factors in the medium of the cell culture system demonstrated that human BM-MSCs secreted significantly more VEGF, bFGF, and angiopoietin-1 than BM-MNCs under normoxia and hypoxia conditions. Excluding angiopoietin-1, hypoxia-induced BM-MSCs and BM-MNCs secreted significantly higher levels of all angiogenic. To relieve pain, 100 mg tramadol hydrochloride was injected intramuscularly under strict aseptic conditions. After 20 minutes, cells suspended in 20 ml NS (contained in 20 injectors (1 ml per injector); cells were then injected intramuscularly into the lower limb (20 sites, 3 cm intervals, 1-1.5 cm depth, and 0.5-1 ml BM-MSCs or BM-MNCs per site). Each foot ulcer received a 2 ml injection of cells, as well as the surrounding subcutaneous tissue. The study period was 24 weeks, while the clinical parameters were as follows: increase in pain-free walking distance, improvement in leg perfusion, ankle-brachial index (ABI), transcutaneous oxygen pressure (TcO_2_), and magnetic resonance angiography (MRA) analysis. Improved clinical outcomes were faster, with the BM-MSC group achieving 100% ulcer healing earlier than the BM-MNC group at six weeks post cell therapy and maintaining the highest throughout clinic visits. By the completion of clinical follow-ups, the BM-MSC group experienced much more extended benefits in ABI and TcO_2_ and collateral vessel formation. The BM-MSC group showed a sustained increased MRA score from baseline till the end of the study. However, there was no obvious difference in pain relief amputation rates. The authors, therefore, concluded that BM-MSC therapy was safer, better tolerated, and more effective than BM-MNCs for promoting lower limb perfusion and foot ulcer healing in diabetic patients with critical limb ischemia. (Table 3).

### 5.3. Cellular Products in Improving Microcirculation and Lowering Amputation Rates

Kirana et al. in an RCT-isolated subjects into Bone Marrow Mesenchymal Stem Cell (BM-MSC) group and expanded bone marrow cells enriched in CD90+ cells (Tissue Repair Cell or TRC) group. The purpose of this study was to assess the efficacy, safety, and viability of bone marrow-derived cellular product transplantation in terms of improving microcirculation and reducing amputation rates in diabetic subjects with diabetic ulcers by inducing revascularization. The study included diabetic subjects with critical limb ischemia, having arrived at a stage of no choice for revascularization intervention or surgery. Parameters to be examined were wound healing, ankle-brachial index and TcPO_2_, reactive hyperemia, and angiographic imaging before and after cell therapy. Patients were followed up for 52 weeks following treatment. Incidentally, the results indicated that in the BM-MSC group, two subjects failed to achieve complete wound healing. At the same time, one among the two died of an unknown cause after receiving a major limb amputation. One subject suffered severe subarachnoid bleeding a few weeks before study completion despite achieving wound healing. In the TRC group, one patient died before achieving complete wound healing and clinical follow-ups due to multiple organ failure following sepsis after forefoot amputation. Surprisingly, subjects undergoing BM-MSC treatment showed much more significantly improved outcomes than TRCs in ulcer-healing rate and ankle-brachial index. However, there was no significant difference in TcPCO_2_. Microcirculation improved in some transplanted patients in both groups. These results lead the authors to conclude that Bone Marrow Mesenchymal Stem Cells (BM-MSCs) and expanded bone marrow cells enriched in CD90+ cells were both safe and feasible [33] (Table 4).

### 5.4. The Potential of Allogeneic Stem Cell Sheets for Treating Diabetic Foot Ulcers

Moon et al. in a randomized clinical trial study aimed at determining the potential of Allogeneic Stem Cell (AD-MSC) sheets for treating diabetic foot ulcers. The allogeneic ASC sheet is a 5 cm hydrogel sheet that contains allogeneic ASCs. Following extraction, ASCs were distinguished by the expression of stromal cell-associated markers such as CD10, CD13, CD29, CD44, and CD90. These cells lacked the expression of hematopoietic stem cell-related markers (CD34 and CD45). The genomic stability of ASCs was assessed using karyotyping. The final product underwent a series of additional efficacy release tests, including cell count confirmation and viability assessment. The study period was twelve weeks. However, those subjects that experienced complete wound healing before the study completion had their treatment halted but continued with normal clinical follow-ups to evaluate long-term safety within the designated study period. Autologous stem cells and allogeneic stem cell sheets were applied directly to the wound bed after debridement improved complete wound closure faster. The rate of wound size reduction at one-week post cell application was higher in the treatment group than in the control group. The treatment group observed almost double the rate of complete wound closure at eight weeks than the control. In the treatment group, further improvement in incomplete wound closure at study completion, while control improved only by a lesser margin in the same period. Outcomes of the Kaplan-Meier median time to complete closure were much earlier in days in the treatment group, while a delay of more than twice several days in the control group was prominent. At study completion in 24 months, this study reported a nonsignificant elevation of antibodies in both the treatment and control groups. Nevertheless, they did not observe any signs of rejection. Regarding these outcomes, the authors, therefore, pointed out that patients with diabetic foot ulcers who do not demonstrate any clinical improvements in wound healing even with optimal blood sugar control possess reasonable vascularity (transcutaneous oxygen pressure > 40 mmHg) and with no obvious signs of infection are most likely to respond well to allogeneic (AD-MSC) therapy [34].

### 5.5. Intralesional Allogeneic Adipose-Derived Mesenchymal Stem Cells

Uzun et al. carried out a randomized-controlled single-blind study to investigate the safety and effects of intralesional allogeneic adipose-derived mesenchymal stem cell (allogeneic AD-MSC) injection in diabetic patients with chronic foot ulcers. The subjects were randomized into the treatment group (allogeneic AD-MSC group) who received a single dose allogeneic AD-MSC intralesional and control group (standard diabetic treatment). Both groups were reported to have undergone a similar wound dressing method and clinical follow-ups after administering cell therapy. Postadministration follow-up parameters for subjects' evaluation included demographics, wound characteristics, wound closure time, amputation rate, and clinical scores. Results indicated that the range of days in the meantime to wound closure was lesser in the AD-MSC group compared to the control group. AD-MSC group recorded higher scores in SF-36 physical functioning, general health parameters, and costs. Surprisingly, both study groups noticed no adverse events during the entire follow-up period. There was a more remarkable improvement in the Wagner grade I subjects than in Wagner grade II. Wound closure was achieved in 85% of lesions in 17/20 patients in the AD-MSC group. With these findings, researchers concluded that in the treatment of reasonably low-grade diabetic foot ulcers with no signs of infection, intralesional injection of allogeneic AD-MSCs was safe and effective, with positive benefits to wound healing [35]. (Table 2).

## 6. DFU Management through Stem Cell Modern Theory

The last decade has seen growing interest in stem cell therapy for diabetic foot ulcers. Stem cells are suggested to use various mechanisms to achieve their intended purpose. The purpose of this research was to search and identify the literature pertaining to the use of stem cells for the treatment of diabetic foot ulcers and analyze their safety and efficacy. Here, we give a comprehensive report on the five selected RCTs. In our final study selection, there were two major origins of the cells employed. Apart from AD-MSC and BM-MSC trials identified in this study, numerous other stem cell sources have been used in the treatment of diabetic foot ulcers [36]. This review paper included two trials that adopted adipose tissue-derived mesenchymal stem cells and three trials that used bone marrow-derived mesenchymal stem cells. According to the respective studies undertaken, the trials were furthermore partitioned into AD-MSCs 40%, where 20% were allogeneic and 20% were autologous stem cells. In comparison, the bone marrow-derived covered 60% of where 30% is mononuclear cells, 15% is mesenchymal stem cells, and 15% is an autologous stem cells (AB-MSCs). The modes of cell administration applied included intramuscular injection into the ischemic limb, injection around the ulcer, and direct application of cells onto the wound bed. Primary endpoints varied from one trial to another. All included RCTs noted significant improvement in wound healing and general clinical outcomes after stem cell therapy, with no records indicating any severe adverse complications resulting from stem cell therapy. Stem cell therapy rectifies the fundamental pathophysiology of the diabetic foot ulcer. These cells take advantage of their potential to secret growth factors and cytokines that promote angiogenesis and collagen remodeling, which results in the formation of conducive habitats for the wound [37].

Mesenchymal stem cells are the most preferable and prospective cell source for the treatment of various diseases due to their capacity to differentiate into osteoblasts, adipocytes, chondrocytes, and cardiomyocytes. It is worth noting that the movement of stem cells is quite complex and is governed by an infinite range of cytokines, adhesion molecules, and essential growth factors.

### 6.1. Mechanisms Employed by Mesenchymal Stem Cells

There are two main mechanisms MSCs use to aid in repairing damaged tissue. Mesenchymal stem cells have the potential to renew, replace, or repair the necrotic tissue by directly differentiating into effective cells to blend with damaged ones. The potential of several secret factors enables stem cells to adhere to other distinct cell types on the unit in the regeneration cycle. These elements in secretion could either be paracrine or exocrine in nature, making them flexible to weigh in and effective in protecting functional cells from apoptosis. These factors suggestively activate and mobilize endogenous stem cells to reside in areas of tissue injury. Based on their source of origin, the in vitro and in vivo characteristics of mesenchymal stem cells display divergence, and they tend to behave dissimilarly in response to the local microenvironment. Immunoregulation arbitrated by mesenchymal stem cells runs across the coordination of cell contact-dependent mechanisms and soluble factors. Functional amendments of T-cells, B-cells, dendritic cells, monocytes/macrophages, and natural killer cells are related to the immunoregulatory potential of mesenchymal stem cells. Mesenchymal-secreted cytokines are reported to be associated with the interaction between MSCs, monocytes, and regulatory T-cells (Tregs). However, evidence points out the fact that mesenchymal stem cells utilize cell-to-cell communication and do not necessarily rely on metabolic balance [37]. Mesenchymal stem cells are reported to be involved in all three phases of a wound due to their potential to migrate to injury sites. They accelerate wound healing via immune regulation and growth factor production, which in turn strengthen neovascularization and reepithelialization, restore angiogenesis, and promote resulting in the promotion of wound closure [38].

### 6.2. The Principle of Stem Cells

The critical factor of physiological wound healing is the new blood vessel formation within the transitional tissue that fills in the wound in the process of healing by a secondary intention, herein referred to as granulation tissue. Vasculogenesis and angiogenesis are the two processes leading to the granulation of tissue. Neovascularization of granulation tissue is a process that precedes angiogenesis, vasculogenesis, or both. Endothelial cell migration and proliferation activate the development of new capillaries from the prevailing vascular structure known as angiogenesis [39]. Furthermore, angiogenesis is attainable by introducing growth factors like stem cells that have endothelial progenitor cells (EPCs) and mesenchymal cells as mature proteins and complementary DNA-carrying vector systems (cDNA-plasmid).

### 6.3. Characteristics of Stem Cells

Characteristics of mesenchymal stem cells include the potential to self-renew and display differentiation into multiple tissue-forming cell lineages, namely, osteoblasts, adipocytes, chondrocytes, tenocytes, and myocytes. Additionally, stem cells are noted to express surface CD markers comprising CD44+, CD73+, CD90+, and CD105. Moreover, the lack of CD34, CD45, CD14, and HLA-DR makes MSCs dissimilar from hematopoietic cells [39]. On the other hand, through paracrine signaling, MSCs also have regenerative, regulative, and regulatory effects indicative of a considerable therapeutic capability [40].

### 6.4. Applicability of AD-MSCs and BM-MSCs

Adipose-derived mesenchymal stem cells and bone marrow-derived mesenchymal stem cell surface markers are complementary in nature, notably, CD10, CD13, CD29, CD44, CD54, CD71, CD90, CD105, CD106, CD17, and ASTRO-1. They are negative for hematopoietic lineage markers CD45, CD14, CD16, CD56, CD61, CD62E, CD104, and CD106, including for the endothelial cell (EC) markers CD31, CD144, and Von Willebrand factor, which are harmful to AD-MSCs. Under favorable conditions, AD-MSCs acquire the potential to stimulate angiogenesis, secrete growth factors, and differentiate into multiple lineages [36]. Consequently, they are capable of arousing human dermal fibroblast proliferation by directly engaging cells and paracrine stimulation in the reepithelialization phase of wound healing. As a result, AD-MSCs are suggested to be playing a crucial role in the proliferation and migration of cells. In vitro studies have noted that this ability is basically dependent on the number of stem cells administered. In both preclinical and clinical trials, BM-MSCs and AD-MSCs are used for similar purposes based on patients' tolerance, easy accessibility, minimal inessives for extraction, and ethical limitations, while most clinical trials prefer adipose-derived mesenchymal stem cells to bone marrow-derived mesenchymal stem cells. As expected, AD-MSCs are also conveniently transferable [41].

## 7. Limitations and Recommendations

To fully determine the efficacy of stem cell therapy, it is obligatory to consider the limitations of the included studies. The follow-up period in the respective studies was considerably short, making it challenging to establish the long-term effect of stem cell therapy for this purpose. There was quite a limited number of randomized-controlled trials acquired for this review paper. The selected publications had a significantly small sample size. There was no conventional method of administration among the selected journals, meaning that the methods were all individual research dependent, making it hard to come up with tangible conclusions.

Despite achieving significant advances in this field, there has been no convincing published data in line with strictly monitored multicenter RCTs with the conventional method of a cell application. Henceforth, more research needs to be devoted to coming up with consented efforts to realize this necessity.

## 8. Conclusions

Stem cell therapy has been used as adjuvant therapy in the treatment of diabetic foot ulcers. We did not notice any significant differences in the healing effects between AD-MSCs and BM-MSCs. Furthermore, the results portrayed in this study clearly demonstrate that the application of mesenchymal stem cells is safe and efficacious with no clinically visible adverse effects and may therefore help prevent major limb amputations in uncomplicated diabetic foot ulcers. However, there are still several challenges with consensus on the most applicable cell type, route of administration, and dosages. These are some of the significantly prevailing hindrances in conventionalizing the use of stem cells for this purpose.

## Figures and Tables

**Table 1:** Search strategies for study selection.

Serial no.	Search terms	Limitations	Databases		
			Scopus	PubMed	Web of Science
1	“Diabetic foot”	Title, abstract, and keywords	19,690	13,476	20,488
2	“Diabetic feet”	Title, abstract, and keywords	19,690	216	331
3	“Foot ulcer”	Title, abstract, and keywords	11,308	4,702	7,752
4	#1 OR #2 OR #3	No	23,023	15,057	22,621
5	Diabetic	Title, abstract, and keywords	411,183	832,757	482,810
6	#4 AND #5	No	20,543	13,956	21,090
7	“Stem Cells”	Title, abstract, and keywords	512,169	298,773	474,360
8	“"Progenitor Cells”	Title, abstract, and keywords	79,113	58,392	125,821
9	“Mother Cells”	Title, abstract, and keywords	4,991	1,199	4,511
10	“Colony-Forming Unit^∗^”	Title, abstract, and keywords	62,365	32,525	46,925
11	#7 OR #8 OR #9 OR #10	No	582,140	341,572	562,836
12	#6 AND #11	Open access; year: 2003/01/01 to 2021/09/01; article type: journal, control trial, randomized control trial, and human.	126	105	215

**Table 2:** Selected studies with participants, cells employed, cell origin, cohort size, events, and outcomes.

Author	[31]	[32]	[33]	[34]	[35]
Number of patients participated in stem cell therapy	42	37	22	22	10
Total number of patient	96	41	24	39	20
Age (mean ± SD)	65.01 ± 9.5	64 ± 8.9	69.7 ± 5.6	63.6 ± 12.5	57.3 ± 6.6
Sex (male/female)	78/18	15/22	19/5	27/12	12/8
Condition	Diabetic foot ulcers and others	Diabetic foot ulcers and others	Diabetic foot ulcers	Diabetic foot ulcers	Diabetic foot ulcers
Type of cell	Autologous stem cell AB-MSC	Mesenchymal stem cells BM-MSC 20 participants mononuclear cells BM-MNC 21 participants	Mononuclear cells BM-MNCs BMTRCs	Autologous stem cells AD-MSC	Allogeneic stem cells AD-MSCs
Bone marrow	Adipose tissue	Bone marrow	Bone marrow	Source of cell	Adipose tissue
Purification method	Cells separated and centrifuged	Centrifuged, filtered through a thin membrane washed in phosphate-buffered saline	Automated purification system-fitted with the single-use sterile kit having a rotor for cell washing. Then rinsing with human serum albumin-supplemented normal saline	Cells rinse and centrifuge	Washing with phosphate-buffered, centrifuged, and filtered
Culture medium	No	Mononuclear cell layer 1st. Cultured in alpha-modified essential medium containing 10% autologous serum 2nd. Cultured in alpha-MEM	Iscove's modified Dulbecco's medium (IMDM) with 10% fetal bovine serum, 10% horse serum, 5IM containing hydrocortisone, gentamycin-sulphate, and vancomycin in the medium	DMEM with 0.025% type 1 collagenase for 80 min at 37°C. Stromal vascular fraction cultured in DMEM to obtain enough ASCs. ASCs seeded into hydrogel matrix and cultured for expansion	DMEM with human serum 10%, streptomycin 1% solution, and stable glutamine 1% at 37°C under 5% CO_2_
Mode of administration and volume	Intramuscular injection, 1 ml into the ischemic limb, along post and anterior tibia	2 ml cells, injected in basal of foot ulcer and surrounding tissue	Intramuscular injection in M. gastrocnemius ipsilateral 1 ml each in 3 cm^∗^5 cm^∗^4 cm deep	Allogeneic stem cell sheet put direct on the wound bed	Injected into dermoepidermal junction and homogenously the whole of the wound
Success rate	79% (29/37)	100% (41/41)	82% (18/22)	82% (18/22)	90% (9/10)
Reason for failure	Preexisting severe lymphopenia and thrombocytopenia Death due to coronary heart disease	No failure	Death (multiple organ failure due to sepsis postamputation)	Cellulitis, paresthesia, and uncontrolled diabetes cardiac arrest	Recurrent infections and necrosis leading to minor limb amputations
Adverse complications	Major limb amputation in patients with preexisting severe lymphopenia and thrombocytopenia	No acute or chronic adverse events related to BM-MSC or BM-NSC therapy	BM-MNCs (2 amputations, 1 stroke, and 1 death) BMTRCs (1 death)	(Cellulitis, paresthesia, uncontrolled diabetes, and cardiac arrest, not related to treatment. Baseline clinical and lab changes not clinically meaningful) in both groups	No complications were observed in two years of follow ups
Amputations	Prior treatment; 17 major,1 minor, and 17 index	0	2	0	Minor; 2

**Table 3:** Selected studies on stem cell therapy indicating authors, country, aim, mode of administration, and results.

Author, country	Aim	Mode of administration	Results
[31], Czech Republic	To prevent major limb amputations in patients with critical limb ischemia and foot ulcer	Intramuscular injection,1 ml into the ischemic limb, along post and anterior tibia	(i) Improved (ii) Healed group improvement skin perfusion pressure, laser doppler perfusion pressure *P* = 0.05, and laser doppler perfusion pressure heat value in 90 days (iii) Corresponding toe pressure (increased from 22.66 ± 5.32 mmHg to 25.63 ± 4.32 mmHg) (iv) Brachial index increased from 0.14 ± 0.03 to 0.17 ± 0.03 mmHg (v) No significant difference in Transcapillary pressure of oxygen and neutrophil count (vi) Significant increase in C-reactive protein (*P* < 0.05) in poorly healed limbs 30 days after treatment

[32], China	Selecting the most preferable stem cells for the treatment of diabetic critical limb ischemia and foot ulcer	2 ml cells, injected in basal of foot ulcer and surrounding tissue	(i) Stem cells appeared to show better results with an ulcer healing (*P* = 0.022) (ii) Limb perfusion (*P* = 0.040), ankle-brachial index (*P* = 0.040) (iii) Magnetic resonance angiography analysis (*P* = 0.018), with no significant difference in pain relief and amputation rate

[33], Germany	Access for safety, efficacy, and feasibility of cellular products in improving of microcirculation and lowering of amputation rates	Intramuscular injection in M. gastrocnemius ipsilateral 1 ml each in 3 cm^∗^5 cm^∗^4 cm deep	(i) Improved (ii) BM-MSCs had an 83% ulcer healing rate, improved ankle-brachial index (*P* < 0.10) compared with TRCs at 80% (iii) No significant difference in transcapillary pressure of carbon dioxide microcirculation improved in some patients in both groups

[34], South Korea	To determine the potential of allogeneic stem cell sheets for treating diabetic foot ulcers	Allogeneic stem cell sheet put direct on the wound bed	(i) Improved, complete wound closure faster. Rate of wound size reduction at one-week post cell application. Reduction rate 49.6 ± 25% in the treatment group compared to 23.0 ± 32.2% in control group (*P* = 0.007). Complete wound closure of 73% at 8 weeks with 47% observed in controls (*P* = 0.102) (ii) Further improvement in complete wound closure at 12 weeks in the treatment group while control improved only by 53% in the same period (*P* = 0.053) (iii) Outcomes of the Kaplan-Meier median times to complete closure were 28.5 days within the treatment group, while a delay of 63.0 days in the control group was prominent (*P* = 0.033) (iv) The mean time required for complete closure was 40.8 ± 5.3 days in the treatment group and 57.2 ± 3.9 days in the control group. Antibodies were slightly elevated in 27% of patients without clinical signs at 12 weeks and in 20% of patients of the control group. There were no signs of rejection observed

[35], Turkey	To investigate safety and outcomes after intralesional allogeneic adipose-derived mesenchymal stem cell injection in chronic diabetic foot ulcers	Injected into the dermoepidermal junction and homogenously the whole of the wound	(i) Postadministration follow-up parameters for patients' evaluation included demographics, wound characteristics, wound closure time, amputation rates, and clinical scores. Outcomes mean follow-up duration 48.0 (26-50) months, mean ulcer duration 51.5 ± 18.8 days, and lesion size 24.5 ± 5.5 (ii) Mean time to wound closure was 311.0 ± 10.7, range 22-55days in AD-MSC group and 54.8 ± 15.0 days in control group, *P* = 0.002. Higher scores in of SF-36 physical functioning and general health domains (*P* = 0.017) and higher costs *P* = 0.001 in AD-MSC group compared to the control group at *P* = 0.010 (iii) No adverse events were observed throughout follow ups in the bother study group. Wagner grade I:11 patients (55%) and Wagner grade II: 9 patients 45% (iv) Wound closure was achieved in 85% of lesions, 17/20 patients in the AD-MSC group

**Table 4:** Selected studies on stem cell therapy for diabetic foot ulcer, number of enrolled subjects, dropouts, and endpoints.

Study	Enrolled (*n*)	Participated (*n*)	Dropout and reason	End-point control		End-point experimental	
				LA (%)	CUH (%)	LA (%)	CUH (%)
[31]	96	42	13: death 2°CAD (cont.; *n* = 8, exp.; *n* = 5)	44 (20/46)	56 (26/46)	21 (8/37)	79 (29/37)
[32]	41	37	4 death (cardiopulmonary)	N/A	N/A	0	100% (37/37)
[33]	24	22	2, TRC-not met cell product criteria Control 1 withdrew consent 2 died of CV events 1 major amputation 4 weeks prior to recruitment	0	80% (8/10)	18	83% (10/12)
[34]	59	44	5 Withdrawal of consent Not eligible Adverse effects Protocol violation	—	53% (9/17)	—	83% (18/22)
[35]	20	10	2 died due to other causes	N/A	N/A	0	90 (9/10)

Key: LA: limb amputation. CUH: complete ulcer healing. %: percentage.

## Data Availability

Data are available upon contracting the author.

## References

[1] Yazdanpanah L., Nasiri M., Adarvishi S. (2015). Literature review on the management of diabetic foot ulcer.

[2] Levy N., Gillibrand W. (2019). Management of diabetic foot ulcers in the community: an update. *British Journal of Community Nursing*.

[3] Musuuza J., Sutherland B. L., Kurter S., Balasubramanian P., Bartels C. M., Brennan M. B. (2020). A systematic review of multidisciplinary teams to reduce major amputations for patients with diabetic foot ulcers. *Journal of Vascular Surgery*.

[4] Shu X., Shu S., Tang S. (2018). Efficiency of stem cell based therapy in the treatment of diabetic foot ulcer: a meta-analysis. *Endocrine Journal*.

[5] Moher D., Liberati A., Tetzlaff J., Altman D. G., for the PRISMA Group (2009). Preferred reporting items for systematic reviews and meta-analyses: the PRISMA statement. *BMJ*.

[6] Vuorisalo S., Venermo M., Lepäntalo M. (2009). Treatment of diabetic foot ulcers. *The Journal of Cardiovascular Surgery*.

[7] Piette J. D., Schillinger D., Potter M. B., Heisler M. (2003). Dimensions of patient-provider communication and diabetes self-care in an ethnically diverse population. *Journal of General Internal Medicine*.

[8] Andrews K. L., Houdek M. T., Kiemele L. J. (2015). Wound management of chronic diabetic foot ulcers. *Prosthetics and Orthotics International*.

[9] Kerr E. A., Heisler M., Krein S. L. (2007). Beyond comorbidity counts: how do comorbidity type and severity influence diabetes patients’ treatment priorities and self-management?. *Journal of General Internal Medicine*.

[10] Schaper N. C., Netten J. J., Apelqvist J. (2020). Practical guidelines on the prevention and management of diabetic foot disease (IWGDF 2019 update). *Diabetes/Metabolism Research and Reviews*.

[11] Bus S. A., Lavery L. A., Monteiro-Soares M. (2020). Guidelines on the prevention of foot ulcers in persons with diabetes (IWGDF 2019 update). *Diabetes/Metabolism Research and Reviews*.

[12] Nather A., Cao S., Chen J., Low A. (2018). Prevention of diabetic foot complications. *Singapore Medical Journal*.

[13] Lipsky B. A., Senneville É., Abbas Z. G. (2020). Guidelines on the diagnosis and treatment of foot infection in persons with diabetes (IWGDF 2019 update). *Diabetes/Metabolism Research and Reviews*.

[14] Hilton J. R., Williams D. T., Beuker B., Miller D. R., Harding K. G. (2004). Wound dressings in diabetic foot disease. *Clinical Infectious Diseases*.

[15] de Oliveira A. L. M., Moore Z. (2015). Treatment of the diabetic foot by off-loading: a systematic review. *Journal of Wound Care*.

[16] Alexiadou K., Doupis J. (2012). Management of diabetic foot ulcers. *Diabetes Therapy*.

[17] Frykberg R. G., Banks J. (2016). Management of diabetic foot ulcers: a review. *Federal Practitioner*.

[18] Jung J.-H., Fu X., Yang P. C. (2017). Exosomes generated from iPSC-derivatives: new direction for stem cell therapy in human heart diseases. *Circulation Research*.

[19] Apelqvist J., Bakker K., van Houtum W. H., Nabuurs-Franssen M. H., Schaper N. C., on behalf of the International Working Group on the Diabetic Foot (2000). International consensus and practical guidelines on the management and the prevention of the diabetic foot. *Diabetes/Metabolism Research and Reviews*.

[20] Mohseni S., Aalaa M., Atlasi R., Mohajeri Tehrani M. R., Sanjari M., Amini M. R. (2019). The effectiveness of negative pressure wound therapy as a novel management of diabetic foot ulcers: an overview of systematic reviews. *Journal of Diabetes and Metabolic Disorders*.

[21] Faglia E., Favales F., Aldeghi A. (1996). Adjunctive systemic hyperbaric oxygen therapy in treatment of severe prevalently ischemic diabetic foot ulcer: a randomized study. *Diabetes Care*.

[22] Salama S. E., Eldeeb A. E., Elbarbary A. H., Abdelghany S. E. (2019). Adjuvant hyperbaric oxygen therapy enhances healing of nonischemic diabetic foot ulcers compared with standard wound care alone. *The International Journal of Lower Extremity Wounds*.

[23] Lalieu R. C., Brouwer R. J., Ubbink D. T. (2020). Hyperbaric oxygen therapy for nonischemic diabetic ulcers: a systematic review. *Wound Repair and Regeneration*.

[24] Abidia A., Laden G., Kuhan G. (2003). The role of hyperbaric oxygen therapy in ischaemic diabetic lower extremity ulcers: a double-blind randomised-controlled trial. *European Journal of Vascular and Endovascular Surgery*.

[25] Lazzarini P. A., Jarl G., Gooday C. (2020). Effectiveness of offloading interventions to heal foot ulcers in persons with diabetes: a systematic review. *Diabetes/Metabolism Research and Reviews*.

[26] Cardinal M., Eisenbud D. E., Armstrong D. G. (2009). Serial surgical debridement: a retrospective study on clinical outcomes in chronic lower extremity wounds. *Wound Repair and Regeneration*.

[27] Everett E., Mathioudakis N. (2018). Update on management of diabetic foot ulcers. *Annals of the New York Academy of Sciences*.

[28] Lebrun E., Tomic-Canic M., Kirsner R. S. (2010). The role of surgical debridement in healing of diabetic foot ulcers. *Wound Repair and Regeneration*.

[29] Cañedo-Dorantes L., Cañedo-Ayala M. (2019). Skin acute wound healing: a comprehensive review. *International Journal of Inflammation*.

[30] Bruschettini M., Romantsik O., Moreira A., Ley D., Thébaud B. (2020). Stem cell-based interventions for the prevention of morbidity and mortality following hypoxic-ischaemic encephalopathy in newborn infants. *Cochrane Database of Systematic Reviews.*.

[31] Procházka V., Gumulec J., Jalůvka F. (2010). Cell therapy, a new standard in management of chronic critical limb ischemia and foot ulcer. *Cell Transplantation*.

[32] Lu D., Chen B., Liang Z. (2011). Comparison of bone marrow mesenchymal stem cells with bone marrow-derived mononuclear cells for treatment of diabetic critical limb ischemia and foot ulcer: a double-blind, randomized, controlled trial. *Diabetes Research and Clinical Practice*.

[33] Kirana S., Stratmann B., Prante C. (2012). Autologous stem cell therapy in the treatment of limb ischaemia induced chronic tissue ulcers of diabetic foot patients. *International Journal of Clinical Practice*.

[34] Moon K.-C., Suh H.-S., Kim K.-B. (2019). Potential of allogeneic adipose-derived stem cell–hydrogel complex for treating diabetic foot ulcers. *Diabetes*.

[35] Uzun E., Güney A., Gönen Z. B. (2021). Intralesional allogeneic adipose-derived stem cells application in chronic diabetic foot ulcer: phase I/2 safety study. *Foot and Ankle Surgery*.

[36] Ali-Hassan-Sayegh S., Mirhosseini S. J., Lotfaliani M.-R. (2015). Transplantation of bone marrow stem cells during cardiac surgery. *Asian Cardiovascular and Thoracic Annals*.

[37] Blumberg S. N., Berger A., Hwang L., Pastar I., Warren S. M., Chen W. (2012). The role of stem cells in the treatment of diabetic foot ulcers. *Diabetes Research and Clinical Practice*.

[38] Weiss A. R. R., Dahlke M. H. (2019). Immunomodulation by mesenchymal stem cells (MSCs): mechanisms of action of living, apoptotic, and dead MSCs. *Frontiers in Immunology*.

[39] Dehkordi A. N., Babaheydari F. M., Chehelgerdi M., Dehkordi S. R. (2019). Skin tissue engineering: wound healing based on stem-cell-based therapeutic strategies. *Stem Cell Research & Therapy*.

[40] Dinh T. L., Veves A. (2005). A review of the mechanisms implicated in the pathogenesis of the diabetic foot. *The International Journal of Lower Extremity Wounds*.

[41] Hamou C., Callaghan M. J., Thangarajah H. (2009). Mesenchymal stem cells can participate in ischemic neovascularization. *Plastic and Reconstructive Surgery*.

